# Right pelvic kidney during intersphincteric resection for locally advanced rectal cancer: a case report

**DOI:** 10.1186/s13256-019-2151-3

**Published:** 2019-07-10

**Authors:** Hassan Moaiery, Mohammad Aziz Rasouli

**Affiliations:** 1 Department of Surgery, Faculty of Medicine, Kurdistan University of Medical Sciences, Sanandaj, Iran; 2 Clinical Research Development, Kowsar Hospital, Kurdistan University of Medical Sciences, Sanandaj, Iran

**Keywords:** Pelvic kidney, Rectal cancer, Intersphincteric resection

## Abstract

**Background:**

Simultaneous occurrence of colorectal malignancy with pelvic kidney has been considered a rare phenomenon. A review of the related literature revealed three previous reports of rectal cancer and pelvice kidney.

**Case presentation:**

This case report describe the case of 40-year-old Asian man with complaints of bleeding and a feeling of discomfort in his anus. A colonoscopy revealed a raised large multilobulated mass in his rectum. Multiple biopsies of the lesion were done after detecting a tumor in his rectum 4 cm above the dentate line; a diagnosis of rectal adenocarcinoma was made by pathological examinations. Subsequent investigations, carried out by computed tomography (CT) scans, incidentally showed an ectopic pelvic kidney. Because of the progress of the rectal cancer, our patient was a candidate for neoadjuvant radiotherapy. Six weeks after radiotherapy, he underwent total mesorectal excision (TME) surgery maintaining the ectopic kidney after using a coloanal anastomosis for additional curative surgery. A very low anterior resection surgery was performed to maintain the ectopic kidney. Thereafter, adjuvant chemotherapy was performed.

**Conclusions:**

Due to the proximity of the tumor to the pelvic viscera, especially the ectopic kidney, the probability of inadequate abscission of the lesion in surgery and radiotherapy, as well as complications and localized relapse were increased so that the kidney could be maintained. Carrying out careful pre-treatment examinations can result in maintaining an ectopic kidney and its daily conditioned function dependent on the status of the patient, including the proximity of the ectopic kidney to the tumor, anatomical position, and prior damage. The lesson learned from the present case is that radiotherapy and surgery are possible treatments in the presence of pelvic kidney and rectal cancer without incurring renal damage.

**Electronic supplementary material:**

The online version of this article (https://doi.org/10.1186/s13256-019-2151-3) contains supplementary material, which is available to authorized users.

## Background

Kidney development is a complex process that begins during the sixth to eighth weeks of life. Failure of ascent of the kidney will cause the kidney to remain in the pelvis, that is, pelvic kidney [1]. This condition is rare, with an incidence of 1 in every 2100 cases according to reports [2, 3]. The concurrence of ectopic pelvic kidney and rectal cancer is a rare phenomenon. The ectopic kidney is thought to be no more susceptible to disease than the normally positioned kidney [4]. The best strategy for dealing with conditions that are present in two pelvic pathologies at the same time is not clear to clinicians; however, survival of the patient and the elimination of rectal cancer are top priorities. In difficult conditions, such as the existence of another vital organ along with the main pathology, the main treatment is a major challenge. The purpose of this report is to describe the conditions for maintaining healthy tissue despite the close proximity of cancerous tissue in the pelvic floor area with the precise design of radiotherapy and surgery. Here we portray our experience treating an uncommon patient in whom pelvic kidney existed together with rectal disease. We also provide a brief literature review.

## Case presentation

Our patient is a 40-year-old Asian man with complaints of bleeding and discomfort in his anus of 2 months’ duration. He was an employee with average income who did not smoke tobacco or drink alcohol. He had no weight loss or urinary symptoms, and no substantial family history. He denied any significant medical or surgical history. His abdomen was soft, non-tender, and non-distended, with normoactive bowel sounds. In examination, a mass could be touched by finger tips. The mass was large and bleeding. In subsequent examinations, blood was detected in a stool sample. His vital signs were: blood pressure, 130.77 mm Hg; respiratory rate, 18 breaths/minute; heart rate, 83 beats/minute; and temperature within normal limits. Oxygen saturation was 98% on room air on admission. In colonoscopy, a large lobular tumor was diagnosed at 4 cm above the dentate line, which was suspicious for malignancy. Various samples were taken from the tumor. The rest of his large intestine did not show a clear pathologic lesion in the colonoscopy.

High-grade adenocarcinoma was reported in pathological examinations. In subsequent diagnostic procedures, his carcinoembryonic antigen (CEA) level was normal. Computed tomography (CT) scans revealed that metastatic lesions were not detected in his liver, abdominal viscera, and chest. In CT scans with or without contrast, and magnetic resonance imaging (MRI) scanning, an ectopic kidney was detected incidentally on his right pelvis without any prior urinary symptoms. The left kidney was in its original location, and both kidneys were functional. Renal function tests provided normal results.

**Fig. 1:**
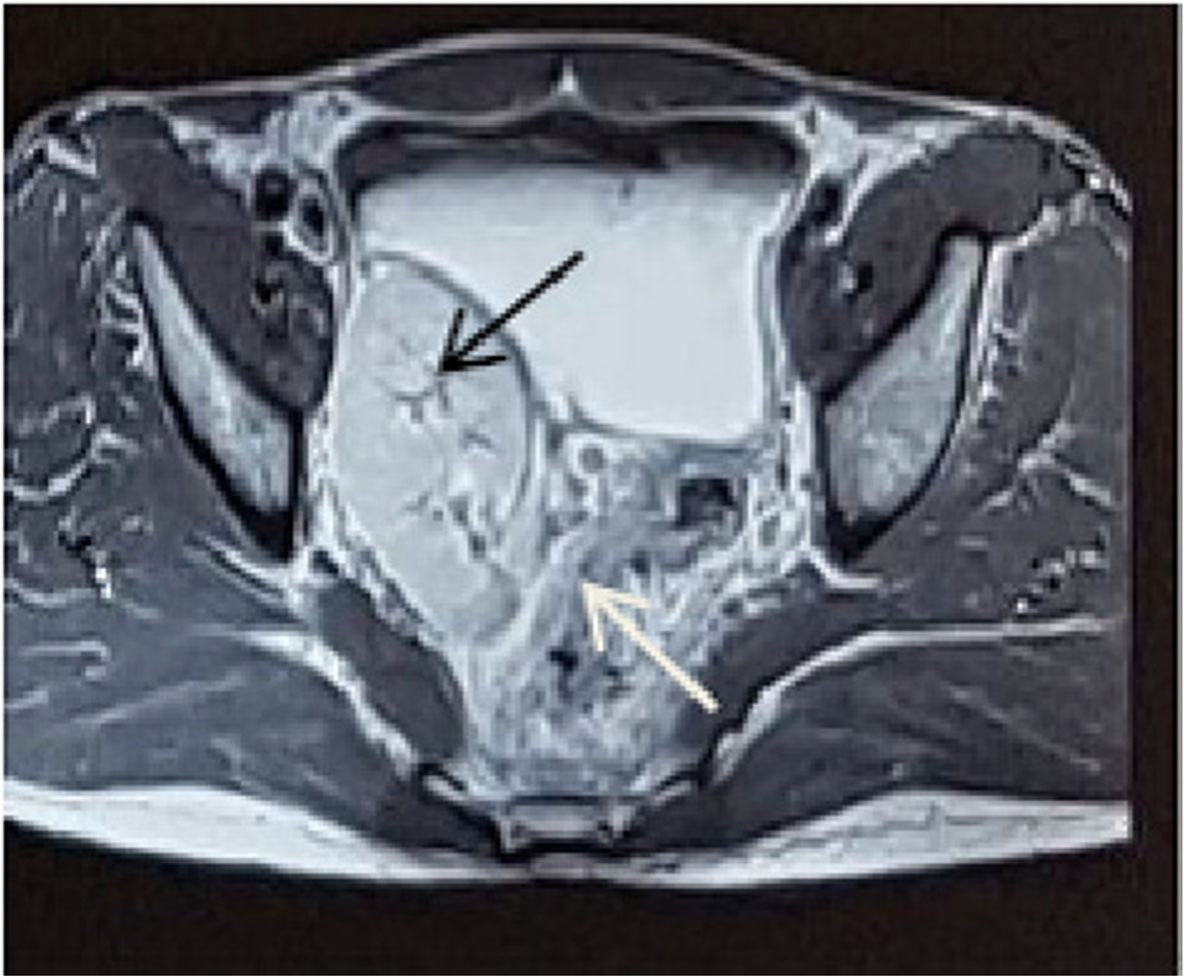
Pelvic-abdominal MRI showing the right kidney in the iliac fossa. Axial view (Black arrow is right pelvic kidney and white arrow is rectal tumor)

**Fig. 2:**
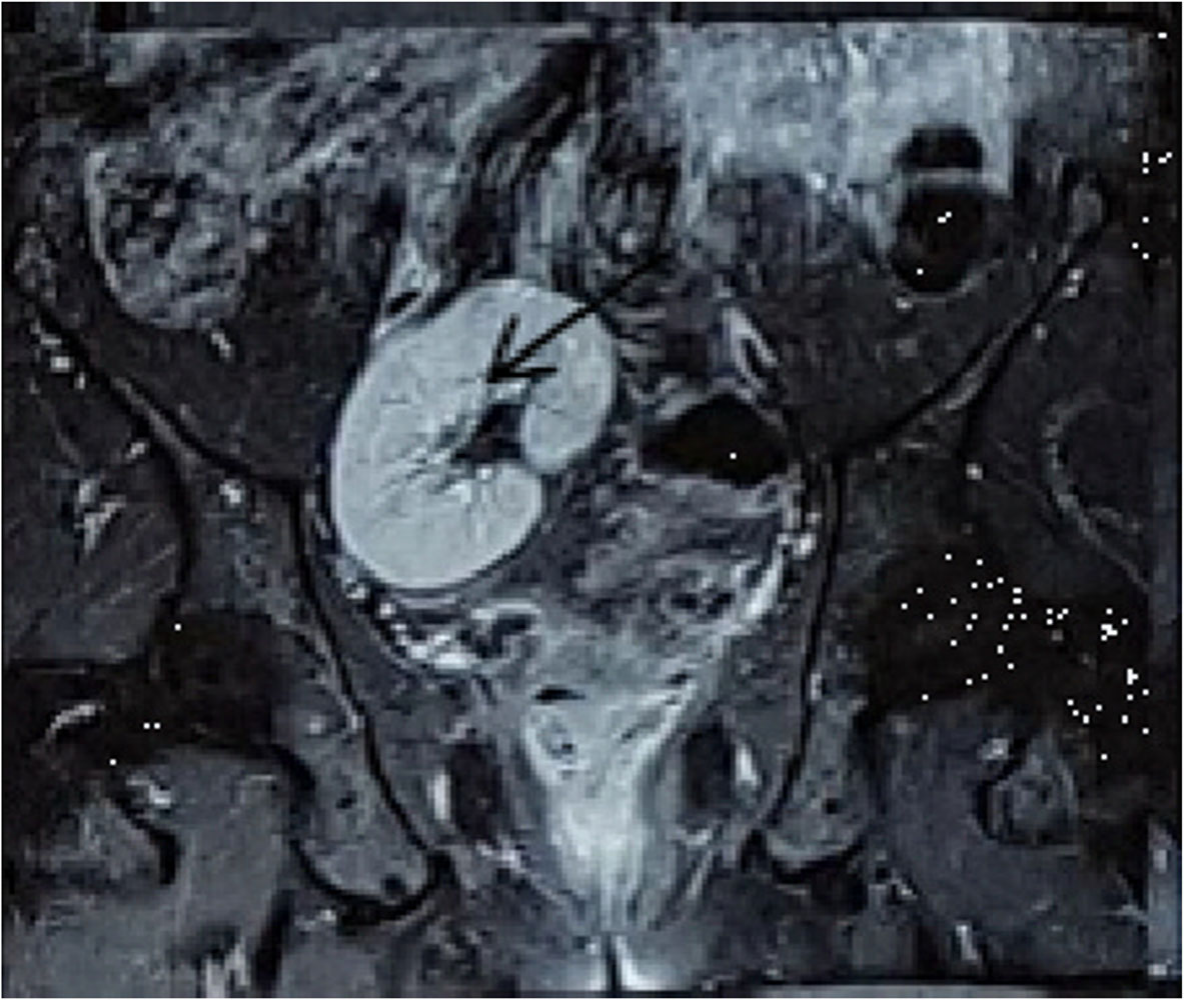
Pelvic-abdominal MRI showing the right kidney in the iliac fossa. Coronal view (Black arrow is right pelvic kidney)

**Fig. 3:**
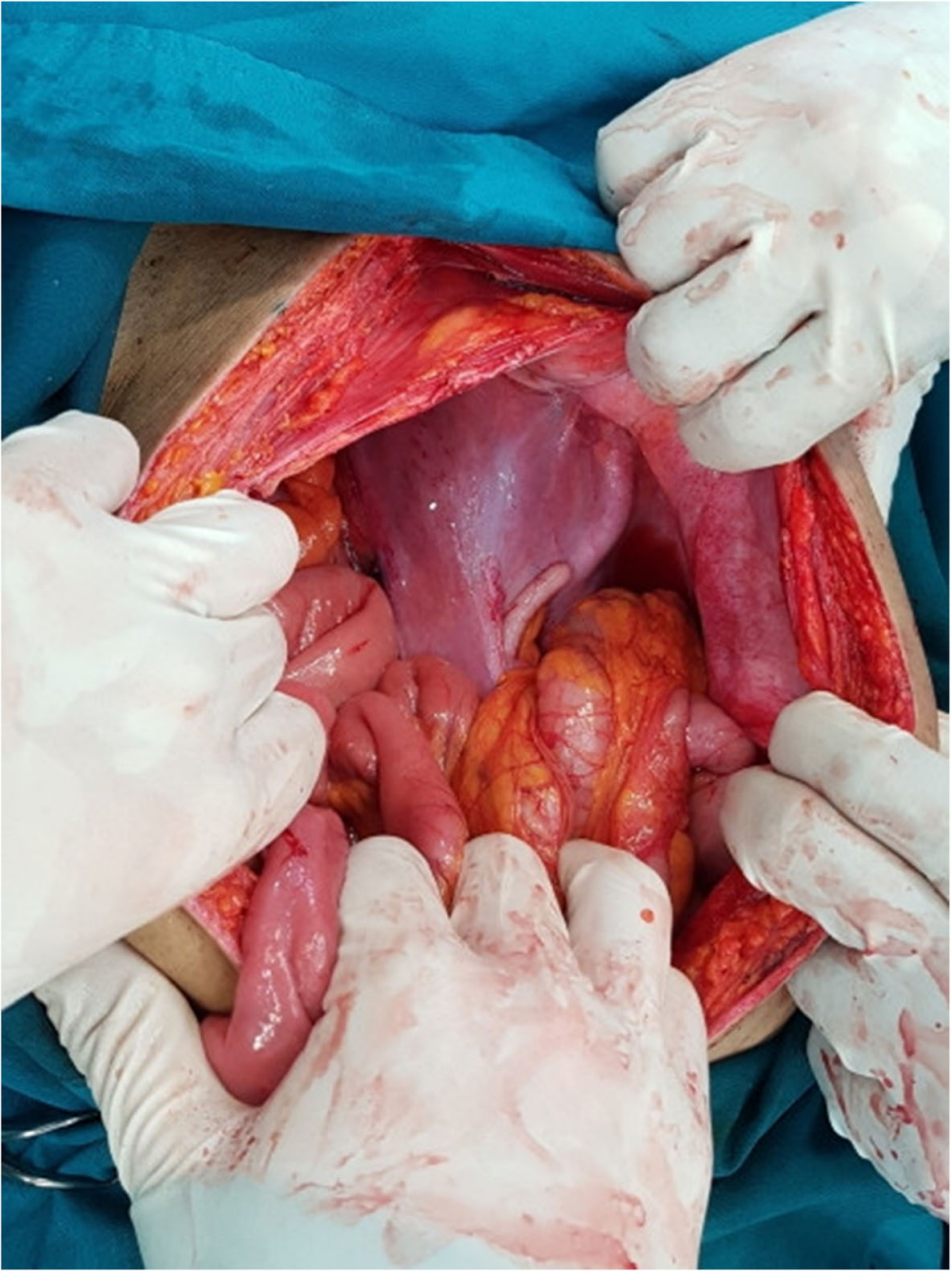
Pelvic kidney in laparotomy view

In subsequent investigations done by MRI scanning for staging the tumor, a pelvic rectum tumor was reported to be interfering with the T3 N1 mesorectal lymph nodes (Figs. 1, 2). The case was discussed in a multidisciplinary cancer team; afterward, our patient was regarded as a candidate for neoadjuvant radiotherapy. He underwent 45 GY radiation in 25 fractions to the pelvis along with capecitabine. He underwent total mesorectal excision (TME) surgery to maintain the ectopic kidney 6 weeks later. After abdominal exploration, his abdominal viscera were examined. There was no metastatic lesion in his liver and abdomen (Fig. 3).

**Fig. 4:**
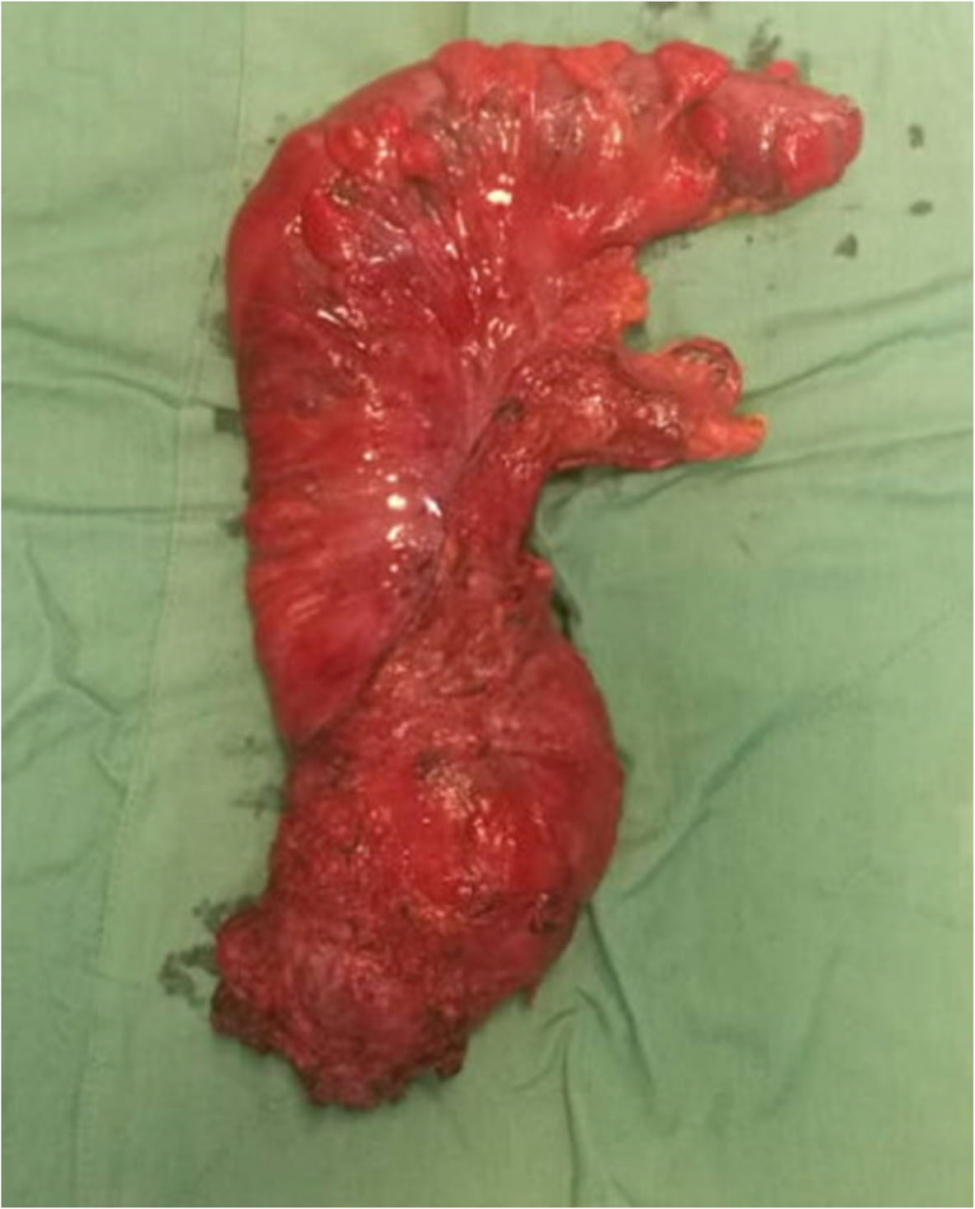
Specimen-orientated surgery of abdomenoperineal resection with TME (left view)

After mobilization of the left colon and the splenic flexure, and the closure of the inferior mesenteric artery (IMA), in the avascular plane, the mesorectum was separated from the fascia propria, and the mesorectal lymph nodes and hemorrhoidal vessels in the anterior and pelvic nerves were fully mobilized, and the distal rectum was removed by an appropriate margin (Fig. 4).

His right kidney was completely inside the pelvis, and while the kidney was carefully protected by the retractor, an attempt was made to minimize the damage to the ectopic kidney because there was a possibility of damage to the pelvic nerve and nephrectomy.

The blood of the right kidney appeared to be supplied by the right superior iliac artery. During the surgery, hematuria occurred to our patient, which was resolved by hydrating him. Then, coloanal anastomosis and temporary ileostomy were performed on our patient. He was transferred to our intensive care unit (ICU). He underwent laparotomy again due to anastomosis leakage a week following the surgery. As a result, a colostomy was performed. Postoperatively, after the reappearance of symptoms, stabilization, and healing of the wounds, he was referred to medical oncology and started adjuvant chemotherapy with 5-fluorouracil, folinic acid, and oxaliplatin (FOLFOX). Follow-up testing (for a year) included routine medical history and physical examination (every 3–6 months), blood tests such as serum CEA, colonoscopy, and radiologic imaging. He was dissatisfied with the permanent colostomy after the end of the treatment. However, the satisfying result was that his kidney was preserved (Additional file 1). Before the surgery, the potential risks and damage to his ectopic kidney and the possibility of its removal were explained to our patient and his consent was obtained. His general condition is appropriate after 1 year and his quality of life has been reported to be satisfying despite the permanent colostomy.

## Discussion

The present case is a 40-year-old Asian man with no previous history of malignant disease; he was referred to our clinic for complaints of rectal bleeding and discomfort in his anus. He was examined. Rectal cancer was diagnosed along with ectopic kidney on the right side of his pelvic cavity. This posed a major challenge in the treatment of radiation and cancer surgery because maintaining the ectopic kidney was part of the treatment plan. The present case report is an attempt to suggest considering a primary rectal cancer as a possible rectal cancer, which can be treated under radiotherapy in the vicinity of a pelvic kidney. The surgical treatment can be performed without causing damage to the kidney. This study aims to share this experience with colleagues working in radiotherapy and surgery departments, and hopes to be beneficial to rectal cancer management.

Although many patients with pelvic kidney are asymptomatic, some of them show complications of hydronephrosis or genitourinary system abnormalities [3]. The pelvic kidney is a result of renal failure during the fetal period. Various theories have been provided about its possible causes, such as growth retardation of the ureteric buds, genetic factors, and maternal factors [3].

Rectal cancer is one of the most challenging issues for cancer surgeons. Locoregional recurrence following proctectomy is highly related to surgical techniques and can cause various complications and morbidities for the patient [5]. These challenges have led to the development of standard techniques, including the TME, designed with regard to pelvic anatomical conditions and rectal cancer biology. In these methods, sharp dissection and the use of anatomical planes and removal of the mesorectal en bloc and preservation of pelvic nerves are of great importance.

In the case presented, in addition to the anatomical difference between the male and female pelvis, with its own specific problems, the kidney position inside the pelvis adds to the difficulties of the mesorectum and tumor in the pelvis [6]. Due to the complexity of the pelvic anatomy and its surroundings, and the presence of the ectopic kidney inside the pelvis, the use of advanced imaging techniques such as MRI, and multidisciplinary team (MDT) surgery procedure design is very important. The tumor should be removed completely to prevent the possibility of future relapse as much as possible. The design of neoadjuvant radiotherapy should be of extra accuracy to expose radiation exactly on the tumor and not on the kidney.

In a review of the literature in PubMed, three cases of rectal cancer were reported with ectopic kidneys, two among whom had an ectopic kidney on the left side and one on the right side [3, 7, 8]. For the patient with the ectopic kidney on the right, the renal artery had been separated from the IMA, which led to the anastomosis of the right renal artery to the IMA. Meanwhile, in our case, the right renal artery was isolated from the IMA and no anastomosis was observed; so, the kidney was maintained inside the pelvis with minimal trauma and without damage to the kidney and arterial and venous system. Considering the presence of only a few cases with rectal cancer with a pelvic kidney [3, 7], we found this case worthy to share.

## Conclusion

The lesson learned from the present case is that radiotherapy and surgery are possible treatments in the presence of pelvic kidney and rectal cancer without incurring renal damage.

## Additional file

Additional file 1: Timeline of the case since diagnosis, neoadjuant chemoradiotherapy, surgery, anastomosis leakage to discharge. (JPG 53 kb)

## Data Availability

All references may be accessed via hyperlink. No datasets were used in the preparation of this manuscript.

## Abbreviations

CEA: Carcinoembryonic antigen
CT: Computed tomography
FOLFOX: 5-Fluorouracil, folinic acid, and oxaliplatin
ICU: Intensive care unit
IMA: Inferior mesenteric artery
MDT: Multidisciplinary team
TME: Total mesorectal excision
