# Supplementary figures and images for: Right pelvic kidney during intersphincteric resection for locally advanced rectal cancer: a case report
Source: J Med Case Rep. 2019 Jul 10;13:210. doi: 10.1186/s13256-019-2151-3 (PMC6617744; doi:10.1186/s13256-019-2151-3)

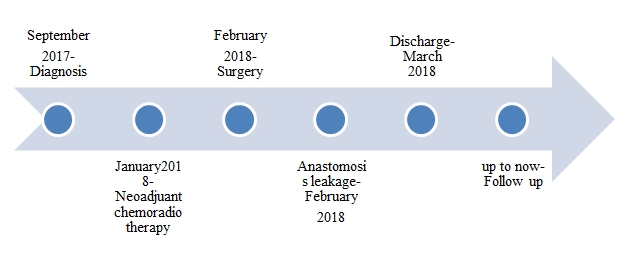

Supplement: Supplementary file 1 — Timeline of the case since diagnosis, neoadjuant chemoradiotherapy, surgery, anastomosis leakage to discharge. (JPG 53 kb) [file 13256_2019_2151_MOESM1_ESM.jpg]
